# Deciphering spatial scales of connectivity in a subsidy-dependent coastal ecosystem

**DOI:** 10.1038/s42003-025-08354-8

**Published:** 2025-06-23

**Authors:** Kyle A. Emery, Jenifer E. Dugan, Robert J. Miller, David M. Hubbard, Jessica R. Madden, Kyle C. Cavanaugh

**Affiliations:** 1 Marine Science Institute, University of California, Santa Barbara, CA USA; 2 Department of Geography, University of California, Los Angeles, CA USA

**Keywords:** Ecology, Biooceanography

## Abstract

Cross-ecosystem subsidies influence the structure and dynamics of recipient ecosystems and can be sensitive to disturbance. Primary production exported from marine to shoreline ecosystems is among the largest known cross-ecosystem subsidies. However, the spatial scales at which this important connection is manifested are largely unquantified. We used local and regional observations of nearshore kelp canopy biomass and beach kelp wrack inputs to evaluate the scales at which connectivity between kelp forests and beaches is maximized. Regardless of the spatial and temporal scales considered, connectivity was highly local (<10 km) and strongest in winter. Kelp canopy biomass was the primary driver of wrack subsidies, but recipient ecosystem attributes, particularly beach width and orientation, were also important. These drivers of connectivity highlight that disturbance to either ecosystem will have large implications for beach ecosystem productivity. Spatial connectivity can regulate recovery from disturbances such that ecosystem connections must be considered in conservation efforts.

## Abstract (web summary)

Spatial connectivity between kelp forests and sandy beaches, in terms of kelp subsidies, is highly localized and primarily dependent on kelp canopy biomass. However, attributes of the beach including width and orientation are also important.

## Introduction

Connectivity within and across ecosystems is a dynamic process that greatly influences populations, communities, food webs, and ecosystem functions^1^. Material exchanges, primarily of organic and inorganic resources, are facilitated and controlled by the degree of ecosystem connectivity and have important implications for food web support and key ecosystem processes, including primary production and decomposition^2–4^. Spatial scaling is important and, in some settings, the size of landscape patches or ecosystems determines the likelihood of connectivity. For instance, larger patches or ecosystems are more likely to be connected with other patches or ecosystems^5^. In other systems, connectivity is driven by the geographic distance between patches or ecosystems^6^. The mechanisms by which resources are exchanged, including physical and biotic processes, also shape the spatial scales of connectivity. Connectivity within and across ecosystems is subject to growing disruptions from climate change and anthropogenic disturbances^6^. For example, warming is reducing terrestrial connectivity by fragmenting lower elevation alpine habitats^7^ and marine connectivity through shortened pelagic larval durations^8^. Quantifying spatial and temporal patterns of ecosystem connectivity and the mechanisms behind these patterns is essential for assessing the resilience of ecosystems to disturbance.

Cross-ecosystem subsidies, whereby a resource is transported from one ecosystem (the donor) to another (the recipient), are one example of ecosystem connectivity. Organic matter exchange across ecosystem boundaries can be facilitated by animal foraging and movement, or by physical processes such as air and water currents^9–11^. These flows can be unidirectional^12^ or reciprocal^13^. Globally, across terrestrial, freshwater, and marine ecosystems, the amounts of observed organic matter subsidies span eight orders of magnitude, ranging from 10^−3^ to 10^5 ^g C m^−2^ year^−1^ ^14^. In many settings, donor ecosystems export organic matter and nutrients, and the recipient ecosystem response is largely controlled by the amount of these subsidies received^15^.

Subsidies can support bottom-up food web processes that structure the recipient ecosystem community, provide energy to higher trophic levels, create habitat, and stimulate biogeochemical processes^16–18^. All of these processes are controlled by the quantity and quality of the subsidy^19^. Subsidies can be dynamic in space and time due to changes in three factors: the productivity of donor ecosystems, the transport of material between donor and recipient ecosystems, and the receptivity of the donor ecosystem. Temporally, the effects of subsidies can differ based on the regularity or stochasticity as well as the timing (e.g., season) of inputs^20^. Processes that transport material between ecosystems (e.g., ocean currents, wind patterns, animal migration patterns) are often highly variable in space and time^3,21,22^, and many factors can lead to variability in the receptivity of an ecosystem including changes in extent or interior-to-edge ratio and disturbance frequency or intensity^23,24^. The relative importance of these three factors is unknown for most cross-ecosystem relationships^14^ and is likely more complex than indicated by direct proximity alone^25,26^.

One of the largest observed cross-ecosystem subsidies occurs between kelp forests and sandy beaches^14^. Detrital exports from kelp forests comprise a major component of their net primary production^27^ and provide trophic support to adjacent ecosystems with low in situ primary productivity^28^. Once this drift kelp is cast ashore as wrack, it shapes the entire ecosystem from bottom-up stimulation of the food web^17,29^ to habitat provisioning^30^ and a myriad of other ecosystem functions^18,31^. These important linkages between kelp forests and beaches are under threat given the intensifying disruptions and climate forcing affecting both ecosystems. Warming seas and overgrazing can lead to declines in kelp abundance and productivity^32,33^ while beaches are losing ground to erosion, sea level rise, and coastal development^34,35^. Disturbance to either ecosystem can weaken or break these vital linkages. However, our understanding of the spatial and temporal scales at which a disturbance in one ecosystem would impact the other is very limited. The source of kelp wrack delivered to sandy beaches is largely unknown, therefore, it is not possible to predict the impact of local- to regional-scale kelp forest loss on the delivery of wrack. The receptivity of beach ecosystems is also expected to decline with growing intensity of disturbances associated with rising sea levels and increased wave-driven erosion, particularly along mixed sandy and rocky shorelines^35–37^. Here, we explore the combined effects of wrack supply and spatial scale on kelp forest subsidies to the sandy beach and investigate how beach condition impacts this relationship.

Giant kelp (*Macrocystis pyrifera*) is found in temperate coastal seas worldwide^38^ and is the dominant nearshore kelp species and wrack source along ~1000 km of North American coast extending from Baja California, Mexico to Point Conception, California, USA^17,18^. We monitored giant kelp wrack dynamics in Santa Barbara County, California at two different spatial and temporal scales. The first dataset consisted of monthly surveys for a 6-year period, from 2015 to 2021, of stranded kelp plants along a continuous 25 km stretch of coastline (local dataset). The second dataset included measurements of kelp wrack cover and stranded kelp plants at 24 sites over a larger ~100 km area between autumn 2017 and winter 2018 (regional dataset). We compared these measurements of kelp inputs on beaches to offshore kelp abundance (a measure of donor ecosystem productivity) and beach width, beach orientation, and wave height (measures of recipient ecosystem receptivity and potential kelp removal from nearshore reefs). We hypothesized that kelp subsidies to beaches are tightly coupled to the dynamics of adjacent, nearshore kelp forests and that the strength of this relationship would decline with increasing distance between donor forests and recipient beaches. We expected physical attributes of the recipient ecosystem to exert control over the amount of kelp wrack deposited and retained by beaches. Finally, we expected this relationship to change with season as the dynamics of both ecosystems are strongly seasonal. We found that connectivity between kelp forest and sandy beach ecosystems is a highly local phenomenon. This relationship is strongest in winter, particularly for wide beaches with proximate kelp forests. The importance of both local kelp supply and physical attributes of the beach ecosystem demonstrates the susceptibility of this cross-ecosystem connection and sandy beach ecosystem functioning to disturbance.

## Results

### Local scale

**Fig. 1:**
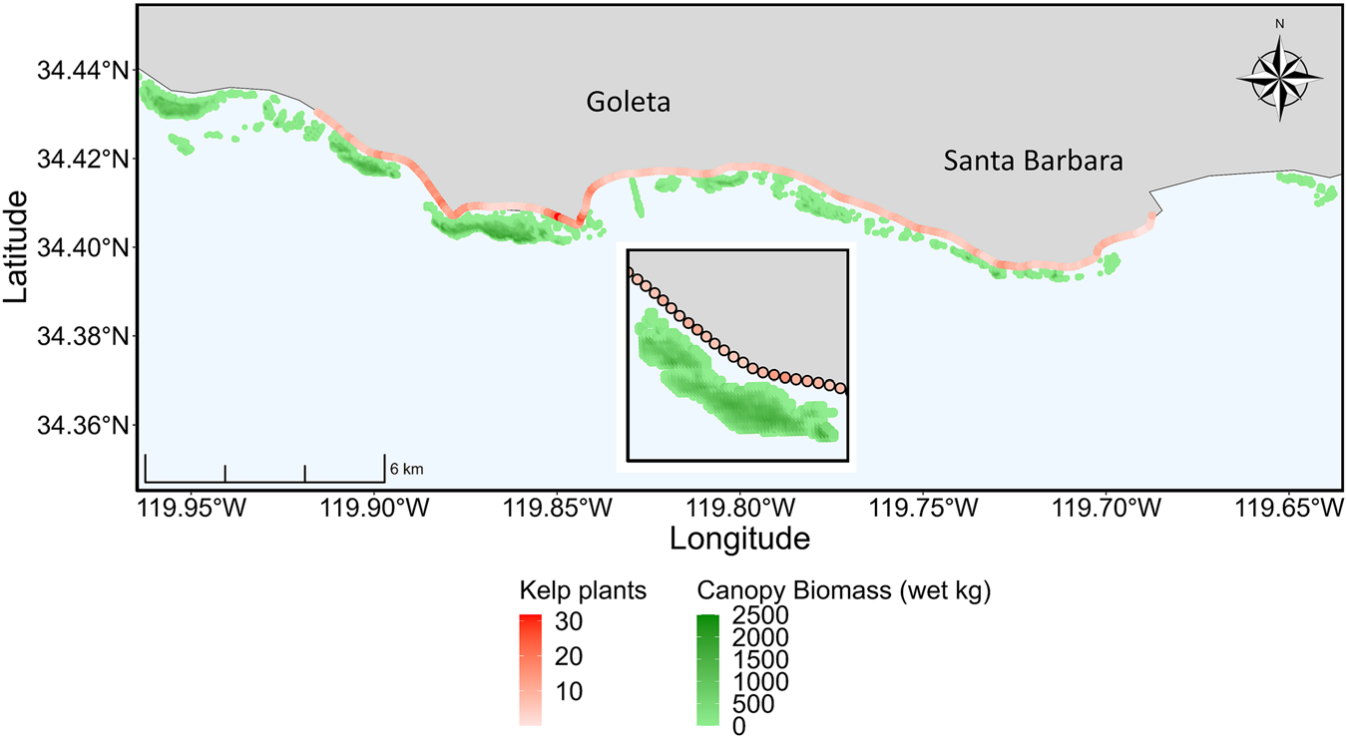
Map of mean kelp plant counts and kelp canopy biomass for the 250 beach segments of the 25 km local dataset. Map of the local scale (25 km) survey region. Kelp plants were counted in 100 m segments which are represented by circular points along the coastline and scaled by the average number of kelp plants counted per segment over the duration of the time series. Average kelp forest canopy biomass for the period of interest is represented by green shading along the coastline and is scaled by average pixel (30 m × 30 m) biomass. Inset shows a representative stretch of coastline providing a more detailed view of the beach segments and kelp forest canopy.

The number of stranded kelp plants, which were defined as a holdfast with at least one 1-meter-long frond (a stipe with blades), observed on beaches varied widely across space and time (Fig. 1). Across the six-year time series, the total number of kelp plants counted monthly in each 100 m segment of 25 km of beach ranged from 0 to 1127 plants and from 39 to 17,197 plants across all 250 segments. Average kelp plant counts for segments ranged from 0.05 ± 0.3 (SD) to 32 ± 140 kelp plants month^−1^ with an average total monthly count of 1900 ± 2721 kelp plants. Observed dry beach widths by segment varied from 0 to 127 m and averaged from 0.1 to 15.0 m. The orientation of the beach segments varied from 87° to 237° but did not vary over time.

**Fig. 2:**
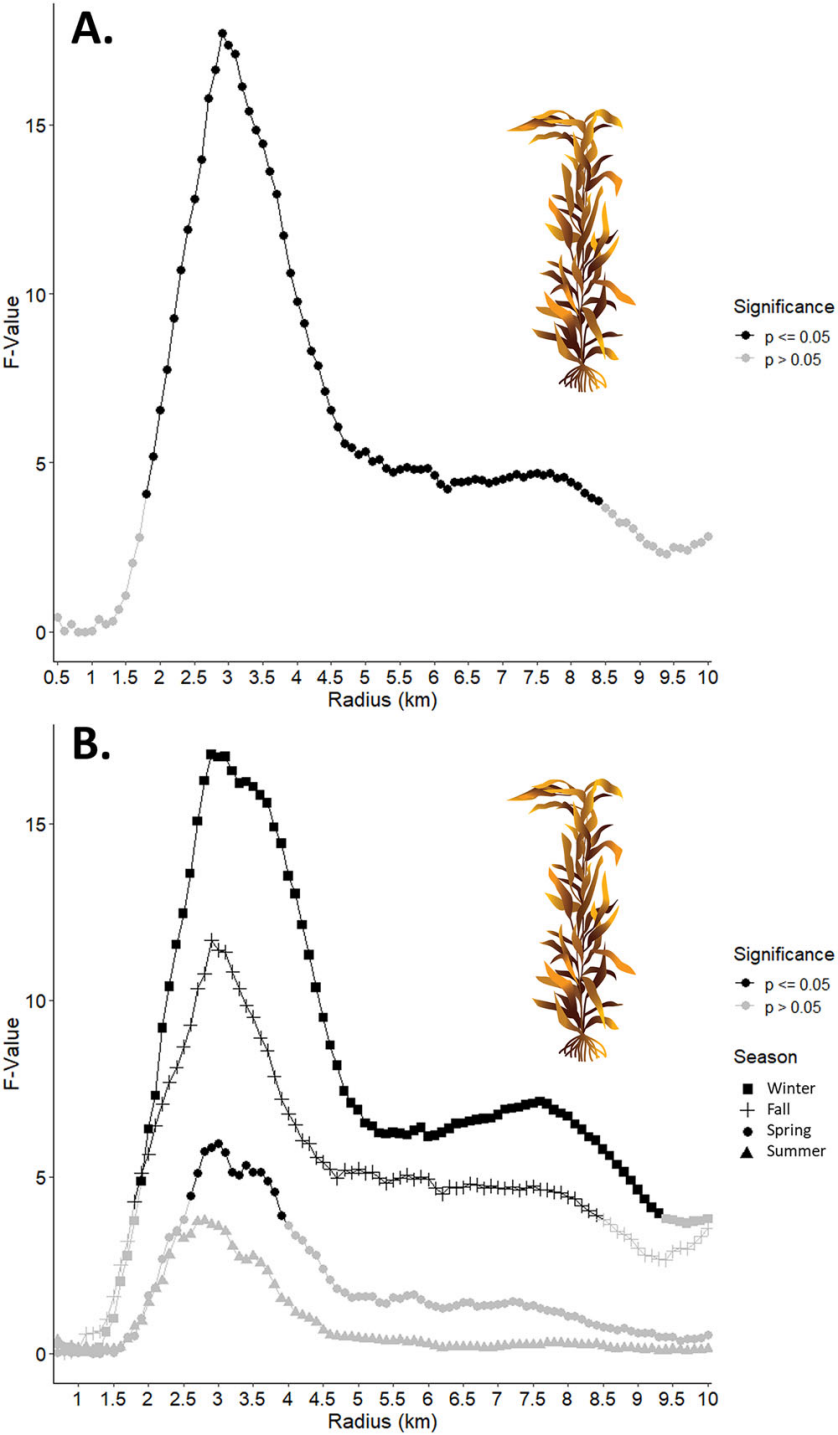
Total and seasonal relationships between kelp plant counts and kelp canopy biomass at increasing radii from the beach segment. The relationship between local scale 25 km plant counts and kelp canopy biomass was modeled over increasing distances from each beach segment for the overall dataset and by season, winter (January through March), fall (October through December), spring (April through June), and summer (July through August). This figure depicts the strength of this relationship (as GLS model F-values) as a function of increasing radii. Points in black are significant (*p* < 0.05) and points in gray are not significant (*p* > 0.05). **A** The optimal radius for the overall dataset was 2.9 km. *n* = 250 measures (beach segments) of kelp plants per data point. **B** The optimal radius for winter (squares) was 2.9 km, fall (+ symbols) was 2.9 km, for spring (circles) was 3.0 km, and for summer (triangles) was 2.8 km (the model for summer was not significant overall). *n* = 250 measures (beach segments) of kelp plants per data point. Kelp illustrations by Monica Pessino, Ocean o’ Graphics, UC Santa Barbara.

The strongest relationship between nearshore kelp canopy biomass and mean abundance of stranded kelp plants occurred when kelp canopy biomass was restricted to a radius of 2.9 km around each 100 m beach segment (i.e., 250 sites) (Fig. 2A, F-value = 17.7, *p* < 0.0001). The strength of the relationship between stranded kelp plants and the kelp canopy radius ascended rapidly to 2.9 km and then quickly declined at greater distances (Fig. 2A). Beyond a radius of 4.5 km the relationship remained relatively unchanged but was still statistically significant (Fig. 2A).

Kelp plant deposition was significantly related to nearshore kelp canopy biomass, dry beach width, cosine of the segment orientation, and sine of the segment orientation (Table 1). The direction components of segment orientation, based on the sine and cosine of the segment orientation, indicated that the lowest amount of kelp was deposited on beaches facing directly south (180°) with more deposition on southwest and southeast facing beaches.

**Table 1:** Model results for the drivers of local and regional kelp subsidies

Dataset	Canopy Biomass		Beach Width		Cosine Orientation		Sine Orientation		Wave Height		
	t-value	*p*-value	t-value	*p*-value	t-value	*p*-value	t-value	*p*-value	t-value	*p*-value	
Local All	**3.8**	**0.0002**	**2.2**	**0.03**	**2.7**	**0.007**	**2.2**	**0.03**	1.9	0.06	
Local winter	**3.7**	**0.0003**	**3.3**	**0.001**	**4.4**	**<0.0001**	**2.0**	**0.05**	1.4	0.2	
Local spring	**2.5**	**0.01**	-0.1	0.9	1.7	0.09	1.3	0.2	1.1	0.3	
Local summer	**2.5**	**0.01**	0.5	0.6	0.2	0.9	**3.8**	**0.0002**	**2.2**	**0.03**	
Local fall	**2.8**	**0.006**	1	0.3	1.5	0.1	−0.4	0.7	0.3	0.7	
Regional kelp plants	**2.6**	**0.02**	−0.3	0.8	−0.2	0.9	1.1	0.3	-	-	
Regional kelp wrack	**7.4**	**<0.0001**	-0.4	0.7	1.6	0.1	**−2.3**	**0.04**	-	-	

Results for models predicting stranded kelp plants are presented for the local scale 25 km kelp plant surveys for the entire time series and for each season. Model results are also presented for the regional scale 24 beach sites for kelp plants and kelp wrack. Significant drivers with *p* < 0.05 are in bold.

Seasonally, the average number of kelp plants counted in a 100 m segment varied more than 4-fold with 3 plants in spring (April through June), 6 in winter (January through March), 8 in summer (July through September), and 13 in fall (October through December). The average monthly number of kelp plants across all 250 segments by season was 774 in spring, 1444 in winter, 2062 in summer, and 3187 in fall. Dry beach widths averaged 1.7 m in winter, 4.8 m in spring, 4.9 m in fall, and 7.7 m in summer. The modeled optimal kelp canopy radius was very consistent across seasons (Fig. 2B). The strongest relationship was found during winter at a radius of 2.9 km (F-value = 16.9, *p* < 0.0001) and was followed by fall at 2.9 km (F-value = 11.7, *p* < 0.001), spring at 3.0 km (F-value = 5.9, *p* = 0.02), and summer at 2.8 km (note that this model was nearly significant (F-value = 3.8, *p* = 0.054)).

The importance of the potential drivers of kelp plant deposition on beaches varied across seasons. In winter, kelp forest canopy biomass, dry beach width, and orientation (cosine and sine) were significant drivers of kelp plant deposition along the coastline (Table 1). Fewer kelp plants were deposited on beaches facing directly south and many more plants were deposited on southwest and southeast facing beaches in winter. In spring, only kelp forest canopy biomass was a significant predictor of wrack biomass (Table 1). While the optimum 2.8 km radius for summer was not significant, kelp canopy biomass, sine of the segment orientation, and mean significant wave height were the primary drivers of kelp plant deposition during that season (Table 1). In the summer, the greatest number of kelp plants were deposited on east-facing beaches, intermediate amounts on south-facing beaches, and the lowest number on west-facing beaches. The summer season in this region experiences swells from the south/southeast compared to predominantly west/northwest swells the rest of the year. Lastly, model results for fall, when kelp inputs were greatest, indicated that only kelp forest canopy biomass was a significant driver of kelp plant deposition (Table 1).

### Regional scale

**Fig. 3:**
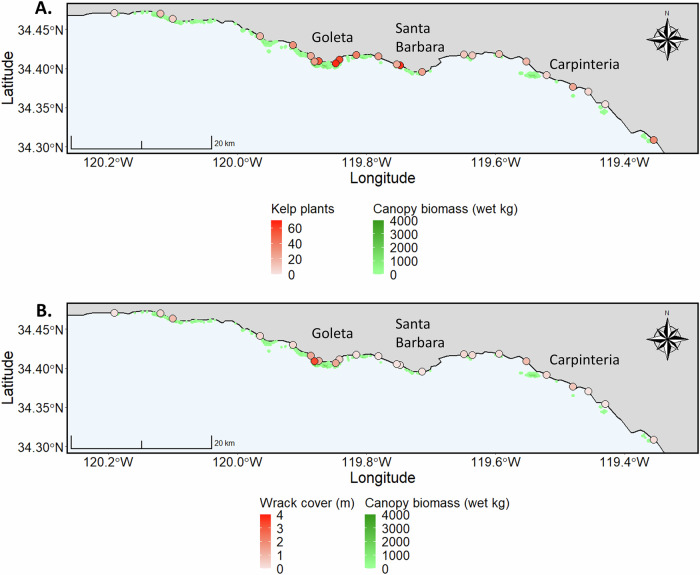
Map of mean kelp plant counts and kelp wrack cover with kelp canopy biomass for the 24 beach sites of the 100 km regional dataset. Map of the 24 regional scale (100 km) beach sites. **A** Kelp plants were counted on 1 km transects at each site and are scaled by the average number of plants counted over three repeat surveys. **B** All kelp wrack including fronds and plants was measured on three replicate transects and is scaled by the average wrack cover. In both panels, average kelp forest canopy biomass for the period of interest is represented by green shading along the coastline and is scaled by average pixel (30 m × 30 m) biomass.

Across the 24 beach sites (spanning ~100 km of coastline), mean kelp plant counts ranged from 2 to 67 plants km^−1^ and averaged 25 plants km^-1^ across all sites (Fig. 3A). The cover of kelp wrack (largely fronds and fragments) ranged from 0.01 to 3.31 m^2^ m^−1^ and averaged 0.52 m^2^ m^−1^ (Fig. 3B). The areal cover of kelp wrack and the number of kelp plants km^−1^ on a beach were not correlated, confirming them as different and independent measures of kelp inputs (*p* = 0.33, Supplementary Fig. 1). Dry beach widths ranged from 4.5 to 32.0 m and averaged 16.3 m while beach orientation varied between 104° and 241°.

**Fig. 4:**
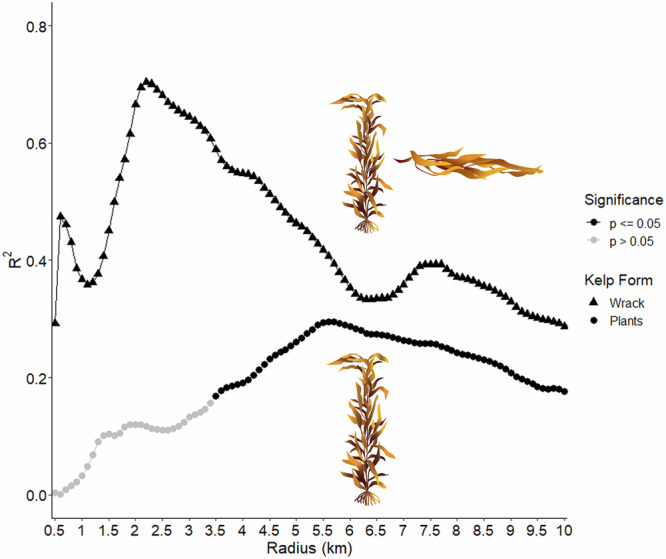
Relationships between kelp wrack cover and kelp plant counts to kelp canopy biomass at increasing radii from the beach sites. The relationship between regional scale kelp plant counts and kelp wrack cover from 24 beach sites and kelp canopy biomass were modeled over increasing distances from each beach. This figure depicts the strength of this relationship (as OLS regression model r^2^ values) as a function of increasing radii where points in black are significant (*p* < 0.05) and points in gray are not significant (*p* > 0.05). The optimal radius for kelp wrack (triangles) was 2.0 km and for kelp plants (squares) was 5.6 km. *n* = 24 measures (beach sites) of kelp wrack and kelp plants per data point. Kelp illustrations by Monica Pessino, Ocean o’ Graphics, UC Santa Barbara.

Analyses of kelp plants and kelp wrack indicated similar, yet slightly different spatial relationships with kelp forest canopy biomass compared to the local scale results. The strongest relationship between nearshore kelp canopy biomass and kelp plants across the regional scale was 5.6 km (Fig. 4, r^2^ = 0.30, *p* = 0.006). The relationship rose steadily to 5.6 km and then gradually declined (Fig. 4). For kelp canopy biomass and kelp wrack cover, the strongest relationship was found at a radius of 2.2 km (Fig. 4, r^2^ = 0.70, *p* < 0.0001). This relationship ascended rapidly to 2.2 km, but then gradually descended such that beyond a radius of 6 km it remained relatively unchanged (Fig. 4).

Kelp plant deposition was significantly driven only by the kelp canopy biomass within 5.6 km and not by dry beach width, orientation, or waves (Table 1). Kelp wrack cover was significantly driven by the kelp canopy biomass within 2.2 km and the sine of beach orientation, but not dry beach width or waves (Table 1). The direction component here indicated that most kelp wrack was deposited on west-facing beaches, intermediate amounts on south-facing beaches, and the least on east-facing beaches. All 24 sites had dry beach during this survey, making beach width less likely to be a limiting factor in kelp plant or kelp wrack deposition. Mean significant wave height was not significant during this time period for kelp plant or kelp wrack deposition and was excluded from these two models following an AIC-based model selection approach.

## Discussion

Given the extent of coastline considered here (25 and 100 km), and the potential for drift kelp to be carried large distances in currents^39^, this key subsidy from nearshore kelp forests to sandy beaches was remarkably local in nature, optimized within a radius of 2.8 to 5.6 km. Although there were large differences in nearshore kelp availability and the spatial scales (100 m segments across 25 km of coast vs beaches distributed along 100 km of coast) and temporal coverage (6 years vs 1 year) of the beach datasets, these factors had minimal impact on estimates of spatial connectivity, pointing to the robustness of this relationship. Our results strongly suggest that local impacts, either negative or restorative, to kelp forests or beaches will alter this connection and subsequently beach community structure and ecosystem functioning^31,40^.

The spatial scale of the connection between kelp forests and kelp deposition on the beach was consistent across seasons, but the connection strength, in terms of deposited kelp biomass, varied considerably, likely due to seasonal variability in both kelp supply and beach receptivity. The relationship between nearshore kelp canopy and kelp plants on the beach was strongest in the winter and weakest in the summer. Due to seasonal erosion from wave disturbance^36,41^, winter is when beaches are generally the least receptive for deposition and retention of kelp subsidies. However, we found that this elevates the importance of wide beaches for maintaining connectivity with proximate kelp forests during that time. The stretches of coastline which retain sand and a dry upper beach year-round are notable as consistent depositional locations in winter, increasing the importance of a local source of kelp. In contrast, during summer when most beaches are wide and more equally receptive to kelp subsidies along the entire stretch of coastline, we observed the least spatially structured connectivity (i.e., lowest spatial variability or consistent depositional patterns along the coast) between kelp forests and beaches. The differences in connectivity between kelp forests and beaches from summer to winter may be informative of future conditions where kelp forests and beaches are more likely to be in a disturbed state.

The local supply of subsidies from kelp forests is a key factor in determining the input of kelp wrack to beaches along the study coast. Kelp biomass is highly variable across space and time^42^, due to environmental factors including substrate availability, disturbance, and nutrient supply^43,44^. Seasonal wave disturbance and subsequent loss of kelp is a primary determinant of seasonal and interannual variability in kelp production and biomass^43^. Other physical factors such as the sedimentation of nearshore reefs can greatly reduce kelp biomass^45^. Biotic factors also have the potential to impact kelp abundance. Changes in species composition due to invasive kelps or other macroalgae can alter biomass dynamics on nearshore reefs^46^. Overfishing at the top of the kelp forest food web can have detrimental cascading impacts on kelp abundance^47^. The supply of kelp can also be dramatically altered and even largely eliminated with overgrazing and the development of persistent urchin barrens^48^. These losses may be exacerbated on a regional scale due to ocean warming and storm disturbance^32^. Our findings quantify at what extent and spatial scales disturbance-driven kelp forest state change would begin to negatively kelp wrack subsidies to beaches.

While local kelp supply was the primary driver of wrack biomass on the beach, we found that the condition of the recipient ecosystem also influenced the linkage between kelp forests and beaches. Physical attributes of sandy beaches vary widely across space and time due to seasonal and long-term changes in sediment supply, erosion, and sea level^35,49^. Two important features that can affect the ability of a beach to receive and retain allochthonous subsidies include width of dry beach habitat and shoreline orientation^36,50^. The presence of dry beach habitat above the reach of the daily high tide level is representative of the area available for kelp deposition^36,41^. We found that the width of dry beach habitat was a significant predictor of kelp plant deposition in the finer resolution local scale dataset, which was largely driven by its significance in the winter season. Importantly, this demonstrates that a sufficient width of dry beach is a necessary factor for kelp subsidies to beaches and its importance in determining where available kelp is stranded varies with season. Our results are also applicable to other types of buoyant materials which can end up distributed along the shoreline including spilled oil, marine debris and microplastics, or stranded pelagic invertebrates and marine mammals.

The receptivity of a beach to wrack subsidies can also be affected by shoreline orientation. A beach facing into the prevailing wind or surface currents is likely to experience greater inputs than a beach where these forces operate predominantly offshore^25,50,51^. The effect of coastline orientation is likely to vary due to seasonal changes in wind and ocean currents and the effect of strong coastline features, such as headlands and embayments, on circulation patterns^26,50,52^. The landscape effect of shoreline orientation and its interaction with current, wave, and wind directions operated differently on subsidy inputs depending on the form of kelp subsidies and the season. The cosine of beach orientation (i.e., the north/south component) was significant for the full local scale dataset, largely due to its importance in the winter season, such that kelp plant deposition was more likely on southwest and southeast facing beaches. Southwest facing beaches in this region are exposed to storm-driven wind and wave action while southeast facing beaches are generally found in the lee of headlands and may experience deposition of kelp due to current recirculation caused by these coastline features. The sine of beach orientation (i.e., the east/west component) was also significant for kelp plant deposition for the full local scale dataset as well as during summer and winter. For the regional dataset, deposition on east-facing beaches was dominant for kelp plants whereas deposition on west-facing beaches was dominant for kelp wrack. Since dry beach width was not limiting during the regional surveys, this difference might be driven by differential transport of the two different kelp wrack forms in the nearshore environment.

The forms of kelp subsidies, wrack (largely fronds and fragments) versus whole plants, differ in their seasonal and disturbance driven patterns of senescence and loss^50,53–55^. We observed a difference in deposition patterns, and significant drivers of deposition, based on the form of the kelp subsidy, with wrack deposition being an even more local-scale process than the deposition of whole plants. This may be driven by variable transport and depositional patterns with respect to the different forms of kelp as they vary in their buoyancy^54,56^ and potential for transport. Drifting whole kelp plants are weighed down by a negatively buoyant holdfast and may be influenced more by deeper currents and travel greater distances, whereas the fronds and fragments which largely comprise wrack cover on the beach generally float on the surface and are influenced more by wind and surface currents^39^. There is also a seasonal component with a greater likelihood of whole plant kelp deposition during late fall and winter in association with their removal from nearshore reefs due to waves and storms while frond and fragment deposition can peak in summer as upwelling-driven spring kelp forest growth begins to senesce^53,55^. Waves are an important driver of kelp removal from nearshore reefs, but the impact of waves varies based on how sheltered or exposed a reef is and the fact that successively stronger wave events are required to dislodge additional kelp as a winter season progresses^57,58^. The importance of waves as a predictor of kelp inputs for the summer seasonal only is likely driven by the different wave dynamics during summer when the predominant wave direction can be from the south/southeast compared to the west/northwest swells the rest of the year^52,59^.

California’s sandy beaches are home to some of the most diverse invertebrate communities in the world and a major component of this diversity is coupled to kelp wrack subsidies^17^. The decline or loss of wrack subsidies to sandy beaches would have far reaching negative impacts on the functioning of this ecosystem^31^. Ecosystem processes, such as nutrient recycling and decomposition would be greatly reduced^60^, limiting fluxes to the adjacent nearshore and/or dune ecosystems^61^. Primary consumer populations would decline in abundance and diversity, with food web effects cascading up to shorebirds and small mammals^17,51,57^. Kelp wrack originating in different locations may vary in its nutritional quality for consumers^62^. Therefore, it is crucial to quantify the important yet precarious connectivity between these two ecosystems as the spatiotemporal variability in kelp wrack deposition likely drives consumer and other dependent species’ populations^57^. Disturbances to either ecosystem can disrupt this critical linkage and based on the local scale of connectivity demonstrated here, the loss of beach receptivity or kelp supply will be highly detrimental. Beach ecosystems face numerous negative impacts from local-scale management and development, such as beach grooming and armoring, which reduce subsidy supply to the beach directly via removal and indirectly via increased reflectivity of the shoreline and loss of dry beach zones^17,40,63^. The receptivity of sandy beaches to these subsidies is declining as sea levels rise, reducing or eliminating the dry upper beach zone^37^. All of these factors have the potential to disrupt kelp forest to beach connectivity, leading to declines in wrack deposition, reduced duration of wrack deposition on the beach, or changes to the proportion of kelp which is deposited on the beach compared to transported offshore. The ability for beach ecosystems to recover from such disturbance events may also be a question of connectivity, dependent on the distance between the beaches which maintain subsidy inputs and ecosystem functioning through seasonal and extreme disturbance events^63^.

Our results show that connectivity between kelp forests and beaches is strongest at local scales, of less than 10 km, and is influenced by both kelp supply and beach condition. Although correlative in nature, our results suggest that long-distance transport of kelp to beaches, although it is not uncommon^38,64,65^, is generally less important than local sources. This implies that management actions affecting either of these factors could impact beach ecosystems. Local impacts on kelp forests caused by processes like urchin grazing are difficult to ameliorate (but see ref. ^66^), although artificial reefs have been used to mitigate kelp losses due to anthropogenic impacts^67^. Beach condition, however, is more amenable to management. Dune restoration^68^, seawall removal^69^, and restoration of sand supplies^70^ can positively affect beach width and therefore kelp wrack deposition. In southern California, remote sensing products like the Landsat-derived kelp biomass used here could help identify promising locations for such actions. Consideration of adjacent ecosystems can extend the benefits of ecosystem restoration beyond the target ecosystem.

## Methods

### Study system

The Santa Barbara Channel is located between the northern Channel Islands and the California mainland within the Southern California Bight in the northeast Pacific Ocean. This is a highly productive region of the coastal ocean which supports abundant giant kelp (*Macrocystis pyrifera*) forests along a mixed coastline of predominantly rocky and sandy shores. The oceanographic climate is characterized by storms in the fall and winter, wind-driven upwelling in the spring, and mild conditions in the summer^52^. Kelp forests and sandy beach ecosystems experience seasonal storm disturbance in the winter which generally removes kelp and erodes the beaches^41,57^. Kelp growth is maximized during spring upwelling conditions^43^, but there is consistent senescence of kelp fronds throughout the year^53^. Dry beach widths typically reach maximums during summer and early fall and minimums during late winter and early spring^41^. Wrack cover on beaches (the abundance of marine macrophyte detritus) is similarly seasonal, with peaks in late summer and early fall^36^. Narrow beaches may be less likely to retain wrack due to removal by waves and spring high tides^36,41^. Large scale climatic events also impact the region with enhanced disturbance of both kelp forests and beaches during El Niño years^36,71^.

### Beach datasets

The analyses conducted in this study utilize three unique datasets which quantify the input of kelp wrack subsidies to beaches. These datasets span different geographic regions, from a local-scale 25 km stretch of coastline to a regional-scale 100 km span of beach sites. They also capture kelp wrack inputs at different spatial resolutions, including 1 m wide cross-shore transects, 100 m wide sections of beach, and 1 km wide sections of beach. Lastly, the datasets have different temporal coverage, including continuous monthly surveys for over five years, repeat surveys during a fall/winter season, and a single snapshot survey during the fall season. These datasets are all complemented with concurrent kelp forest canopy biomass and beach condition data. The vast spatial and temporal coverage of these datasets, captured at multiple different resolutions, provides a powerful tool for estimating the spatiotemporal variability in wrack delivery and its drivers. We utilize these datasets described in detail below in concert to draw important generalizations about the spatial scale of connectivity between donor and recipient ecosystem, if this relationship varies temporally, and what drives the relationship.

### Local data

Whole giant kelp plants, consisting of a holdfast and a minimum of one 1 m long frond (stipe and blades), were counted in 100 m segments along a continuous 25 km stretch of coastline in Santa Barbara County, California, USA (Fig. 1). Surveys were conducted monthly (with few exceptions) from August 2015 to July 2021 for a total of 66 surveys. In addition to kelp plant counts, the width of the dry upper beach was measured for each 100 m segment ever month. Dry beach width was measured from the back beach barrier (e.g., cliff base) to the 24-h high tide strandline which represents the daily total water level (tide + wave set up + wave runup). The shore-normal orientation of each 100 m segment in degrees was calculated in ArcGIS (Esri).

### Regional data

Giant kelp wrack cover and whole giant kelp plant counts were measured at 24 sandy beaches in Santa Barbara and Ventura Counties, California, USA (Fig. 3). Wrack cover was measured once at each site in fall (October or November) 2017 on three replicate transects, running from the upper beach boundary (i.e., cliff base) to the high swash level, using a line-intercept method and averaged^17^. Wrack cover can include whole plants but is largely comprised of fronds and fragments. Whole giant kelp plants were counted on three replicate surveys beginning fall (October or November) 2017 and continuing through winter (February) 2018 along a 1 km stretch of beach centered on the location of the three wrack transects. The first of the three kelp plant counts was conducted on the same day as the wrack cover survey. The two subsequent surveys occurred between the initial survey and February 2018. Dry beach width was measured from the back beach barrier to the 24-h high tide strandline which represents the daily total water level (tide + wave set up + wave runup). Importantly, all sites surveyed in this project had dry beach. The shore-normal orientation of each beach in degrees was calculated in ArcGIS (Esri).

### Kelp forest canopy dataset

Quarterly estimates of giant kelp canopy biomass for 30 × 30 m pixels from Landsat imagery were aggregated for the time periods corresponding to the beach surveys of kelp plants and kelp wrack^72^. To compare to the local dataset, quarterly canopy biomass data from the first quarter of 2015 through the third quarter of 2021 (*n* = 27 quarters) was averaged for each pixel in the study region. To compare to the regional dataset, quarterly canopy biomass data for the year preceding beach sampling, the fourth quarter of 2016 through the third quarter of 2017 (*n* = 4 quarters), were averaged for each pixel for the roughly 100 km stretch of coastline encompassing the 24 sites.

### Wave data

We considered wave effects on kelp wrack and plant inputs using modeled wave data for each site or segment. Wave data, as mean daily significant wave height, (Coastal Data Information Program – Monitoring and Prediction 2024)^73^ were averaged for the different time periods of interest (Sept–Nov 2017 for regional kelp wrack inputs, Sept 2017–Feb 2018 for regional kelp plant inputs, Aug 2015–July 2021 for local kelp plant inputs, and the 4 seasonal time periods within Aug 2015–July 2021 for local seasonal kelp plant inputs). Therefore, each site or segment had an associated mean significant wave height integrated over the study period and representative of the wave disturbance to the area.

### Statistical analyses

For both beach datasets, we analyzed the relationship between kelp wrack cover and/or whole giant kelp plants and the potential supply of nearshore giant kelp using a linear modeling approach. For the local dataset, we averaged the kelp plant count for each of the 250 segments across the full 66-month time series such that each 100 m segment is considered a beach site for this analysis (*n* = 250 sites). We then created buffers of increasing radii from the center of each segment such that each segment had 100 associated buffers of increasing radii (at 0.1 km increments) from 0.1 km to 10.0 km. We then summed mean canopy biomass within each of the 100 buffers for each of the 250 segments. Next, we used linear regression to determine the radius at which average nearshore kelp canopy biomass best predicts the mean number of kelp plants observed in each segment. Because of the inherent spatial autocorrelation between the segments, we utilized general least squares (GLS) regression modeling to analyze this relationship. Specifically, we used an autocorrelation-moving average correlation structure (corARMA) within the GLS model (‘nlme’ R package)^74^ where the autoregressive order (p) and moving average order (q) were both set to 1. To ensure that the spatial autocorrelation was resolved by the model, we compared model residuals with and without the autocorrelation structure using an autocorrelation function (acf in the ‘stats’ R package). The radius corresponding to the best performing model was selected by running ANOVA on the 100 GLS radii models produced by this analysis.

The kelp canopy biomass within this optimum radius for each of the 250 segments was then used as a predictor variable in a GLS model along with segment dry beach width, sine of the segment orientation (east-west component), cosine of the segment orientation (north-south component), and time-averaged mean significant wave height to consider the relative importance of supply and retention-based drivers of kelp plant deposition on the beach. Dry beach width indicates the availability of space for kelp deposition to occur. Coastline orientation describes the direction in which the beach segment faces, and if significant, would indicate a role of nearshore processes including wind direction, wave exposure, and water current direction on both kelp removal from the nearshore reef and beach kelp deposition. The time-averaged mean significant wave height term describes the local wave climate during the study period and indicates the potential for wave-driven removal and subsequent deposition of kelp plants. We used the same correlation structure in this GLS model to account for spatial autocorrelation across the segments and compared model residuals with and without the autocorrelation structure as described above.

We examined the potential impact of seasonality on the relationship between kelp forest canopy biomass and kelp plant counts. The mean number of kelp plants per segment were calculated by taking the mean of all surveys conducted in the winter months of January, February, and March; the spring months of April, May, and June; the summer months of July, August, and September; and the fall months of October, November, and December. We conducted the same GLS model selection with ANOVA as described above for the 100 radii developed for each segment for each season. Kelp canopy biomass derived from Landsat was also partitioned by season for these analyses.

Following identification of the optimal radius of kelp canopy biomass for each season, we ran GLS models using the same variables as described above, except with seasonal mean kelp canopy biomass from within the optimal radius and seasonal mean dry beach width and mean significant wave height. All GLS models for the seasonal analyses were run with the same correlation structure described above and model residuals were compared with and without the autocorrelation structure to ensure that we accounted for the spatial autocorrelation between segments. An AIC-based model selection approach identified the full model as the best fit for the full and seasonal datasets.

For the regional dataset we similarly created buffers of increasing radii from the center of each beach such that each site had 100 associated buffers with increasing radii (at 0.1 km increments) from 0.1 km to 10.0 km. All 100 buffers for each of the 24 beaches were then intersected with the kelp canopy biomass dataset to estimate the total mean canopy biomass contained within each radius. Next, we used Ordinary Least Squares (OLS) linear regression to determine the radius at which average nearshore kelp canopy biomass best predicts the mean number of kelp plants averaged across the three counts and the mean kelp wrack cover averaged across the three transects for each beach. The radii corresponding to the best performing kelp plant and kelp wrack models were selected based on the respective models’ r2 and *p*-values.

The kelp canopy biomass estimates within the two optimum radii identified (for kelp plants and kelp wrack) for the 24 beaches were then used as predictor variables in OLS linear regression models along with dry beach width, sine of the beach orientation (east-west component), cosine of the beach orientation (north-south component), and time-averaged mean significant wave height to consider the relative importance of supply and retention-based drivers of kelp plant and kelp wrack deposition on the beach. An AIC-based model selection approach identified models without the wave height term as the best fit for both kelp plants and kelp wrack.

### Reporting summary

Further information on research design is available in the Nature Portfolio Reporting Summary linked to this article.

## Supplementary information

Supplementary Material

Description of Additional Supplementary Files

Supplementary Data

Reporting Summary

## Data Availability

The data that support the findings of this study are available through the Environmental Data Initiative (10.6073/pasta/c6e6aeefba29bfac28e509ac18db71b9)^75^ and the Supplementary Information Files. All other data are available from the corresponding author on reasonable request.

## Code availability

Code is available from the corresponding author upon reasonable request.
