# Supplementary material for: Deciphering spatial scales of connectivity in a subsidy-dependent coastal ecosystem
Source: Commun Biol. 2025 Jun 23;8:949. doi: 10.1038/s42003-025-08354-8 (PMC12185743; doi:10.1038/s42003-025-08354-8)

Supplementary Information

Title

Deciphering spatial scales of connectivity in a subsidy-dependent coastal ecosystem

Authors

Kyle A. Emery[1,2 *], Jenifer E. Dugan[1], Robert J. Miller[1], David M. Hubbard[1], Jessica R. Madden[1], Kyle C. Cavanaugh[2]

Affiliations

[1] Marine Science Institute, University of California, Santa Barbara, Santa Barbara, CA 93106

[2] Department of Geography, University of California, Los Angeles, Los Angeles, CA 90095

***Corresponding Author**

Kyle A. Emery

Email: emery@ucsb.edu

Supplementary Figure 1: Linear regression between kelp wrack cover ($m^2\ m^{-1}$) and kelp plant deposition (count km^{-1}) for the 24 site regional dataset ($R^2 = 0.04$, $p = 0.33$).

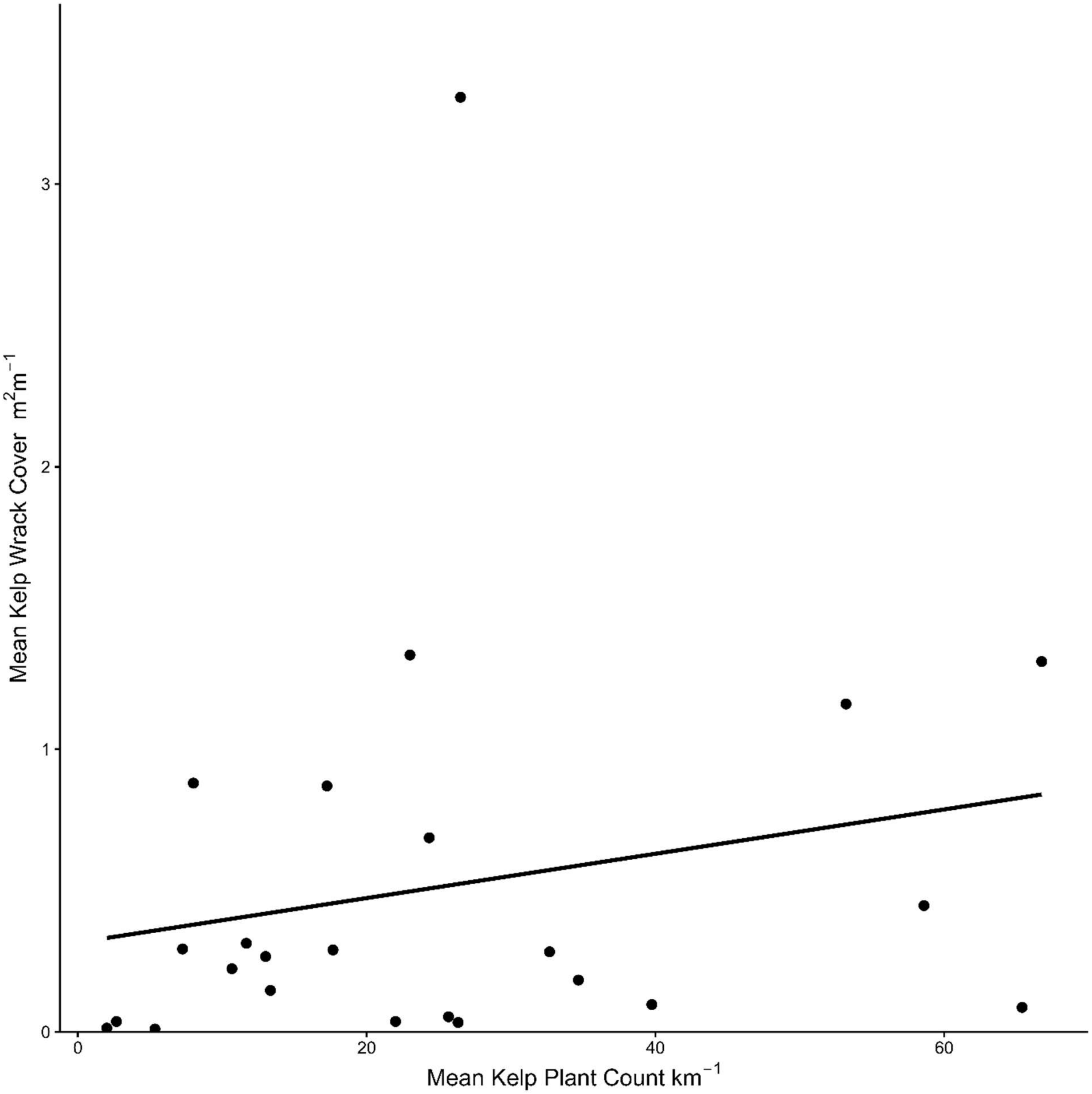

Supplement: Supplementary file 1 — Supplementary Material [file 42003_2025_8354_MOESM1_ESM.pdf]
